# Prostaglandin E1 reduces apoptosis and improves the homing of mesenchymal stem cells in pulmonary arterial hypertension by regulating hypoxia-inducible factor 1 alpha

**DOI:** 10.1186/s13287-022-03011-x

**Published:** 2022-07-16

**Authors:** De-Tian Jiang, Lei Tuo, Xiao Bai, Wei-Dong Bing, Qing-Xi Qu, Xin Zhao, Guang-Min Song, Yan-Wen Bi, Wen-Yu Sun

**Affiliations:** 1 Department of Cardiovascular Surgery, Qilu Hospital (Qingdao), Cheeloo College of Medicine, Shandong University, Qingdao, 266035 Shandong China; 2 Department of Cardiovascular Surgery, Weifang Yidu Central Hospital, Qingzhou, Weifang, 262500 Shandong China; 3 Department of Cardiovascular Surgery, Qilu Hospital, Cheeloo College of Medicine, Shandong University, Jinan, 250062 Shandong China

**Keywords:** Mesenchymal stem cells, Pulmonary arterial hypertrophy, Stem cell homing, Prostaglandin E1, Apoptosis, Hypoxia-inducible factor 1 alpha

## Abstract

**Background:**

Pulmonary arterial hypertension (PAH) is associated with oxidative stress and affects the survival and homing of transplanted mesenchymal stem cells (MSCs) as well as cytokine secretion by the MSCs, thereby altering their therapeutic potential. In this study, we preconditioned the MSCs with prostaglandin E1 (PGE1) and performed in vitro and in vivo cell experiments to evaluate the therapeutic effects of MSCs in rats with PAH.

**Methods:**

We studied the relationship between PGE1 and vascular endothelial growth factor (VEGF) secretion, B-cell lymphoma 2 (Bcl-2) expression, and C-X-C chemokine receptor 4 (CXCR4) expression in MSCs and MSC apoptosis as well as migration through the hypoxia-inducible factor (HIF) pathway in vitro. The experimental rats were randomly divided into five groups: (I) control group, (II) monocrotaline (MCT) group, (III) MCT + non-preconditioned (Non-PC) MSC group, (IV) MCT + PGE1-preconditioned (PGE1-PC) MSC group, and (V) MCT^+PGE1+YC-1-PC^MSC group. We studied methane dicarboxylic aldehyde (MDA) levels, MSC homing to rat lungs, mean pulmonary artery pressure, pulmonary artery systolic pressure, right ventricular hypertrophy index, wall thickness index (%WT), and relative wall area index (%WA) of rat pulmonary arterioles.

**Results:**

Preconditioning with PGE1 increased the protein levels of HIF-1 alpha (HIF-1α) in MSCs, which can reduce MSC apoptosis and increase the protein levels of CXCR4, MSC migration, and vascular endothelial growth factor secretion. Upon injection with ^PGE1-PC^MSCs, the pulmonary artery systolic pressure, mean pulmonary artery pressure, right ventricular hypertrophy index, %WT, and %WA decreased in rats with PAH. ^PGE1-PC^MSCs exhibited better therapeutic effects than ^non-PC^MSCs. Interestingly, lificiguat (YC-1), an inhibitor of the HIF pathway, blocked the effects of PGE1 preconditioning.

**Conclusions:**

Our findings indicate that PGE1 modulates the properties of MSCs by regulating the HIF pathway, providing insights into the mechanism by which PGE1 preconditioning can be used to improve the therapeutic potential of MSCs in PAH.

**Supplementary Information:**

The online version contains supplementary material available at 10.1186/s13287-022-03011-x.

## Background

Pulmonary arterial hypertension (PAH) is a progressive disease characterized by pulmonary vascular remodeling, which leads to increased pulmonary vascular resistance and right ventricle dysfunction [1]. Without treatment, the average duration of survival of patients with PAH was found to be 2.8 years [2]. Furthermore, patients with PAH treated with the appropriate drugs only survived for 7 years [3].

MSCs are multipotent, non-hematopoietic, fibroblast-like adherent cells that can be isolated from different tissue sources, such as the bone marrow, placenta, adipose tissue, dental pulp, and umbilical cord blood. They can differentiate into various types of cells and show specific surface antigen expression. In addition to their ability to differentiate, MSCs exert their therapeutic effects through the secretion of paracrine factors that have angiogenic, anti-apoptotic, anti-inflammatory, and immunomodulatory effects [4].

Stem cell therapy is a novel method for the treatment of PAH [5]. Mesenchymal stem cells (MSCs) serve as novel and effective treatment agents for PAH [6]. In experimental monocrotaline-induced PAH, MSC therapy improved hemodynamics by mitigating lung vascular remodeling [7, 8].

However, at present, there are several limitations to the treatment of PAH using MSCs. Multiple mechanisms in the process of PAH induction can cause severe oxidative stress [9]. Excessive and persistent oxidative stress can reduce the functional potential of MSCs and increase MSC apoptosis [10, 11]. Following their implantation, most MSCs are degraded and eliminated through the liver and spleen, and the number of cells homing to injured tissues is considerably low [12]. MSCs secrete vascular endothelial growth factor (VEGF), which can promote endothelial cell growth and angiogenesis and play an important role in PAH treatment [13]. Thus, the use of interventions that help maximize the therapeutic potential of MSCs has garnered significant interest.

The hypoxia-inducible factor (HIF) pathway is a key pathway in MSCs. More than 1000 genes are directly or indirectly regulated by HIF. Moreover, HIF affects major MSC features including cell viability, proliferation capacity, differentiation, migration pattern, and metabolism [14, 15]. Lificiguat (YC-1) is an inhibitor of the HIF pathway that specifically inhibits HIF-1 alpha (HIF-1α) expression [16].

B-cell lymphoma 2 (Bcl-2) is considered an important anti-apoptosis protein [17, 18]. We studied the degree of apoptosis in MSCs by measuring the protein levels of Bcl-2 and performed flow cytometry to detect the expression of specific cell markers. The stromal cell-derived factor 1 alpha (SDF-1α)/C-X-C chemokine receptor 4 (CXCR4) axis is of significance in MSC migration. The homing of MSCs can be evaluated by analyzing the presence of CXCR4 on the surface of MSCs and the SDF-1α-induced migration of MSCs [19]. In this study, we preconditioned MSCs with prostaglandin E1 (PGE1) and performed in vitro and in vivo cell experiments to evaluate the therapeutic effects of the MSCs in rats with PAH. Our findings showed that preconditioning MSCs with PGE1 improved their homing and reduced MSC apoptosis, thereby improving the overall therapeutic potential of the MSCs in the rat model of PAH.

## Methods

### Isolation, expansion, and labeling of MSCs

MSCs were harvested from the femur of young Wistar rats and anesthetized with 1% pentobarbital sodium. Alpha-Minimum essential medium (α-MEM; Biological Industries, Kibbutz Beit Haemek, Israel) supplemented with 10% fetal bovine serum (FBS; Gibco, Grand Island, NY, USA) was used as the culture medium. The bone marrow was flushed using a culture medium and then cultured at 37 °C in 5% CO_2_. Cell purification was achieved by washing cells with phosphate-buffered saline (PBS) and changing the culture medium after 48 h. Cells from the third to fourth generation were used in the experiments. Before transplantation, the MSCs were labeled with chloromethylbenzamido dialkylcarbocyanine (CM-Dil; BestBio, Shanghai, China) for in vivo cell tracking.

### Preconditioning of MSCs

The MSCs were treated with PGE1 (Sigma-Aldrich, St. Louis, MO, USA) for 24 h. Treatment with YC-1 (MedChemExpress, Monmouth Junction, NJ, USA) was conducted for 12 h before treatment with PGE1. To study the survival and migration of MSCs under oxidative stress, the culture medium was replaced after 24 h of chemical pretreatment, which was followed by treatment with 200 μM H_2_O_2_ for 12 h.

### PAH rat model

All experimental protocols were approved by the Animal Protection Committee of Qilu Hospital of Shandong University. All animals were cared for according to the guidelines for the nursing and use of experimental animals.

Adult male Wistar rats weighing 250 g–300 g (*n* = 48) were used for the experiment. Monocrotaline (MCT) was injected subcutaneously at a dose of 60 mg/kg (MedChemExpress). After 3 days, the CM-Dil-labeled MSCs (10^8^/mL × 0.2 mL) were transplanted by injection through the tail vein. The experimental rats were randomly divided into five groups based on the treatment administered: (I) control group (injected with 0.2 ml PBS), *n* = 6; (II) MCT group (MCT without transplantation of MSCs), *n* = 6; (III) MCT + ^Non-PC^MSC group (MCT and transplantation of non-preconditioned MSCs), *n* = 12; (IV) MCT + ^PGE1-PC^MSC group (MCT and transplantation of PGE1-preconditioned MSCs), *n* = 12; and (V) MCT + ^PGE1+YC-1-PC^MSC group (MCT and transplantation of PGE1 + YC-1-preconditioned MSCs), *n* = 12.

One week after the MSC transplantation, three rats were randomly selected from each group. The blood methane dicarboxylic aldehyde (MDA) and VEGF levels of these rats were measured using an enzyme-linked immunosorbent assay (ELISA).

Four weeks after the transplantation, six rats from groups III, IV, and V were humanely sacrificed for evaluating MSC homing using fluorescence microscopy. At the same time, the systolic blood pressure and mean pulmonary artery pressure of the remaining rats were measured, following which they were sacrificed. Rat hearts were dissected and weighed. The ratio of the weight of the right ventricle to the left ventricle + interventricular septum [RV/(LV + S)] was calculated to evaluate the degree of right ventricular hypertrophy. Lung tissue sections were stained with hematoxylin and eosin (HE). The wall thickness index (%WT) and relative wall area index (%WA) of pulmonary arterioles were calculated.

### Cell surface phenotype analysis

Flow cytometry was performed to determine the phenotype of MSCs. MSCs were washed twice with washing buffer (PBS containing 3% FBS) and incubated for 30 min on ice with the following fluorescein isothiocyanate (FITC)- and phycoerythrin (PE)-conjugated antibodies in the washing buffer: anti-CD29 (1:200,eBioscience, San Diego, CA, USA), anti-CD44 (1:300,eBioscience), anti-CD45 (1:400,eBioscience), anti-CD90 (1:400,eBioscience), anti-CD34 (1:50, Abcam, Cambridge, MA, USA), and anti-CXCR4 (1:50, Abcam) antibodies. The MSCs were rinsed with the washing buffer again, and a FACSCalibur system (BD Biosciences, San Diego, CA, USA) was used for characterizing MSCs. Data were analyzed using the FlowJo software (Treestar, Ashland, OR, USA).

### Western blotting (WB)

Proteins were extracted from the treated cells using an ice-cold lysis buffer containing phenylmethylsulfonyl fluoride. The protein content was determined using a bicinchoninic acid protein assay kit (Beyotime, Shanghai, China) and bovine serum albumin (BSA) as the standard. Proteins were separated using 10% SDS-PAGE and transferred to a PVDF membrane (Millipore Corp., Billerica, MA, USA) using the wet transfer method. Membranes were then blocked for 1 h with 5% skimmed milk or BSA in TBST and incubated overnight at 4 °C with the following primary antibodies: anti-HIF-1α (1:1000; Abcam), anti-CXCR4 (1:1000, Sigma-Aldrich), anti-Bcl-2 (1:200; Cell Signaling Technology, Danvers, MA, USA), and anti-β-tubulin (1:1000; Abcam) antibodies. β-tubulin served as the loading control. The PVDF membranes were then incubated for 1 h with the HRP-conjugated goat anti-rabbit IgG (1:1000; Beyotime). The resulting band intensity was quantified using ImageJ software (Rawak Software Inc., Stuttgart, Germany).

### Apoptosis analysis

An annexin V-FITC/PI apoptosis detection kit (BD Biosciences) was used for evaluating cell apoptosis using flow cytometry.

### Transwell migration assay

Migration assay was performed with a Transwell (Corning Inc., Corning, NY, USA) containing a polycarbonate membrane filter (8 μm pore size). The preconditioned MSCs (200 μL, 1.5 × 10^5^/mL) were cultured onto the upper Transwell chamber, and medium containing SDF-1α (600 μL, 100 ng/mL; PeproTech Inc., Rocky Hill, NJ, USA) was added to the lower Transwell chamber to induce cell migration. After incubation for 12 h, the number of cells in five randomly selected microscopic fields was counted (200× magnification).

### Measurement of MDA and VEGF concentrations using ELISA

Blood samples were collected from rats 1 week after the MSC transplantation. The serum MDA concentration was measured using an ELISA kit (Mlbio, Shanghai, China) to evaluate the oxidative stress levels.

MSCs were pretreated for 24 h, and the culture medium from each well was collected. The VEGF concentration in the medium was measured using an ELISA kit (Abbkine, Wuhan, China).

### Immunofluorescence microscopy

The upper lobe of the right lung of the rats was removed and embedded in an optimal cutting temperature compound (SAKURA, Finetek, CA, USA). Slices (5 μm thick) were cut using a freezing microtome (Leica, Wetzlar, Germany). Nuclei were stained with 4ʹ,6-diamidino-2-phenylindole (Sigma-Aldrich). Stained sections were observed under fluorescence microscopy (Nikon, Tokyo, Japan). The homing ability of MSCs was evaluated based on the frequency of MSCs in lung tissue.

### H&E staining

The left lungs were dehydrated in a graded ethanol series and embedded in paraffin. Slices (5 μm thick) were cut, deparaffinized, and stained with H&E. Sections were observed under an optical microscope (Olympus, Tokyo, Japan).

### Statistical analysis

For data analysis, a one-way analysis of variance was performed, followed by Dunnett’s multiple comparisons test. Differences with *P* < 0.05 were considered statistically significant. Data analyses were performed using SPSS 19.0 (IBM, Armonk, NY, USA).

## Results

### Characterization of MSCs cultured in vitro

**Fig. 1:**
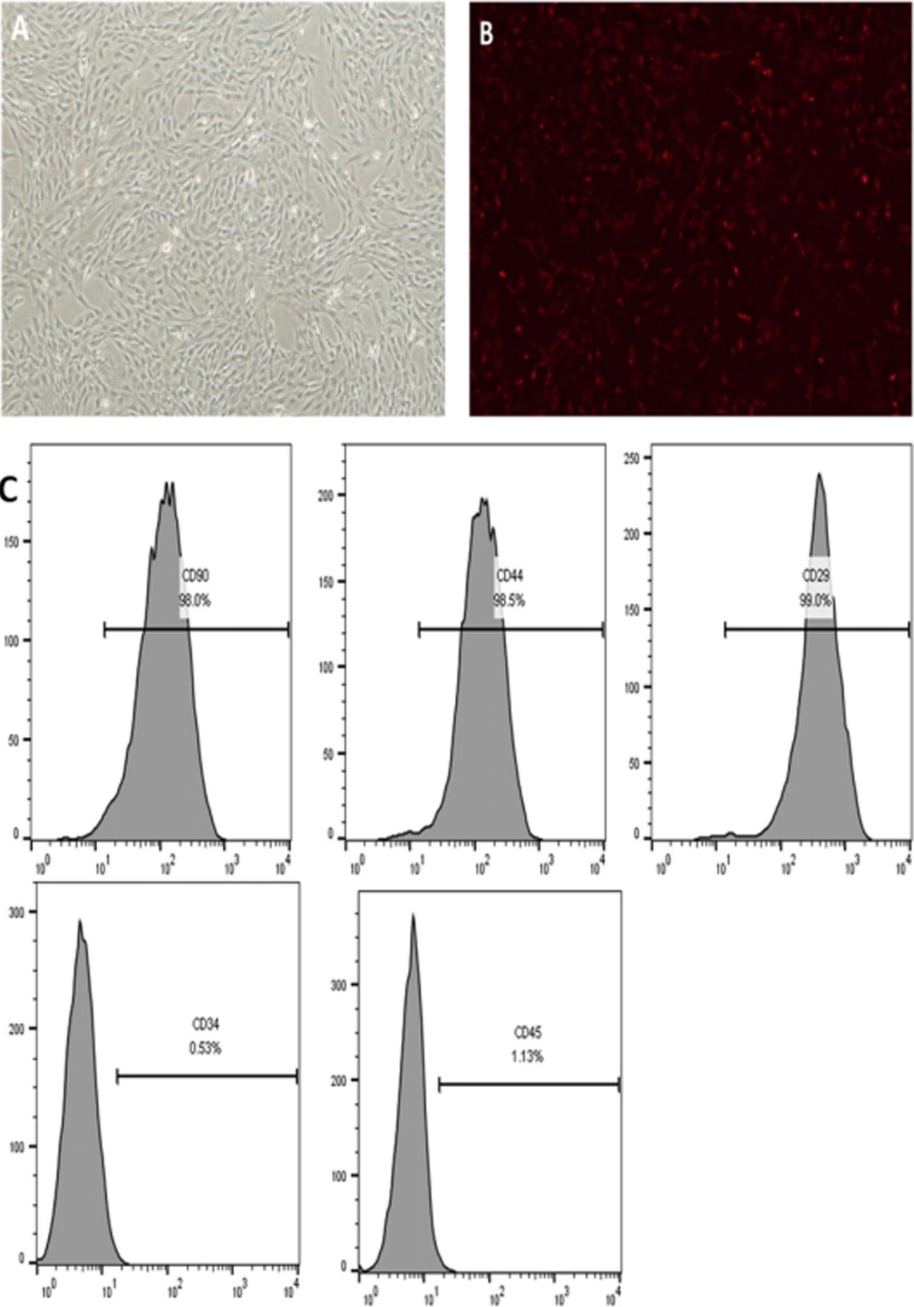
Characterization of MSCs. **A** MSCs exhibited a flat fibroblast-like morphology. **B** Chloromethylbenzamido dialkylcarbocyanine-labeled MSCs emitted red fluorescence. **C** Cultured MSCs were analyzed using flow cytometry and anti-CD90, anti-CD44, anti-CD29, anti-CD34, and anti-CD45 antibodies. *MSC* mesenchymal stem cell

In vitro-cultured third-passage MSCs exhibited a uniform fibroblast-like morphology (Fig. 1A). Most CM-Dil-labeled MSCs emitted red fluorescence as observed under a fluorescence microscope (Fig. 1B). These cells tested negative for CD34 and CD45 but positive for CD90, CD44, and CD29 in the flow cytometry experiment (Fig. 1C). Therefore, the phenotype of the cell population used in our study was consistent with that in other studies [19, 20].

### Relationship among PGE1 administered at different concentrations and VEGF secretion, Bcl-2 expression, and CXCR4 expression in MSCs

**Fig. 2:**
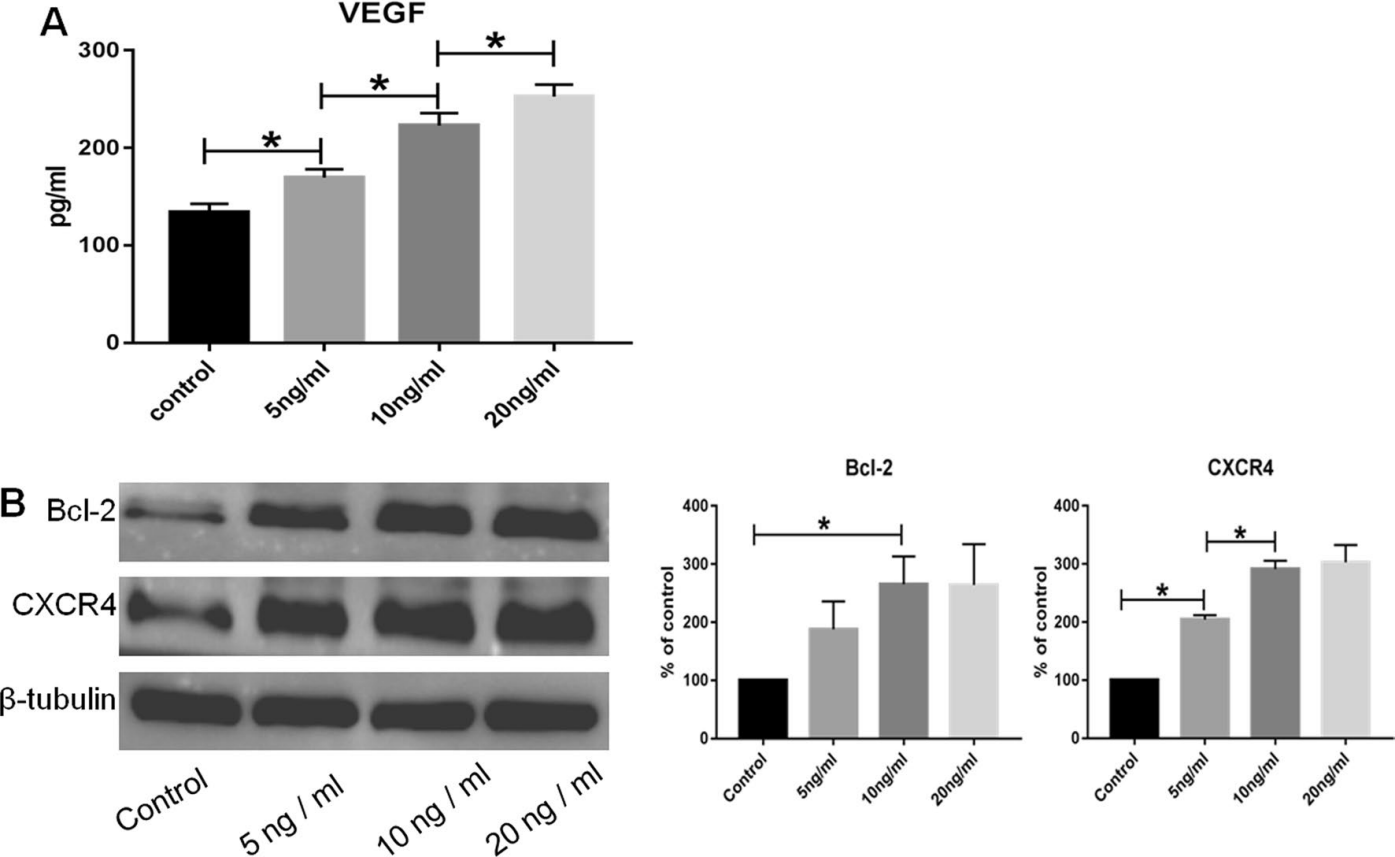
**A** The PGE1-induced VEGF secretion from MSCs was proportional to the PGE1 concentration. **B** PGE1 treatment increased the protein levels of Bcl-2 and CXCR4 in MSCs, with the strongest effect observed upon treatment with 10 ng/mL PGE1 (*P* < 0.05). *Bcl-2* B-cell lymphoma 2, *CXCR4* C-X-C chemokine receptor 4, *MSC* mesenchymal stem cell, *PGE1* prostaglandin E1, *VEGF* vascular endothelial growth factor

The results of ELISA experiments showed that PGE1 (administered at different concentrations) promoted VEGF secretion from the MSCs. VEGF secretion was proportional to the PGE1 concentration and increased persistently with the increase in PGE1 concentration (Fig. 2A). The results of WB experiments showed that with the increase in PGE1 concentration, Bcl-2 and CXCR4 expression in MSCs increased. The effect was the strongest when 10 ng/mL PGE1 was administered but less pronounced when 20 ng/mL PGE1 was used (Fig. 2B). Therefore, 10 ng/mL PGE1 was used in the subsequent experiments.

### PGE1 inhibits H_2_O_2_-induced MSC apoptosis through the HIF-1α pathway

**Fig. 3:**
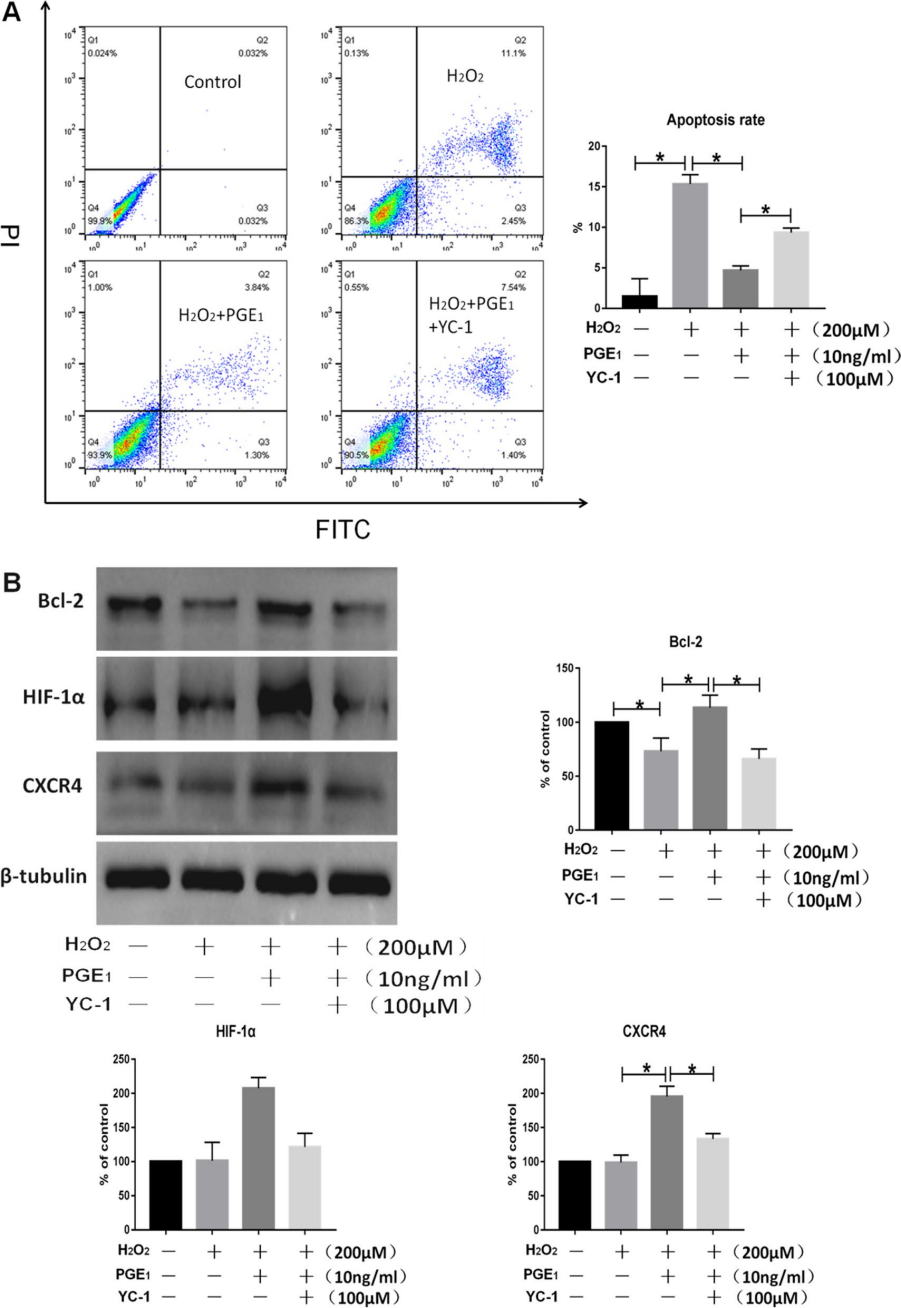
**A** Results of the flow cytometry experiment showed that MSC apoptosis increased in response to treatment with H_2_O_2_. PGE1 treatment reduced MSC apoptosis, whereas YC-1 blocked the protective effect of PGE1. **B** Results of the western blotting experiment showed that the protein levels of Bcl-2 in MSCs decreased in response to treatment with H_2_O_2_. Moreover, PGE1 increased the protein levels of HIF-1α, Bcl-2, and CXCR4, and YC-1 reversed the effects of PGE1 (*P* < 0.05). *Bcl-2* B-cell lymphoma 2, *CXCR4* C-X-C chemokine receptor 4, *HIF-1α* hypoxia-inducible factor 1 alpha, *MSC* mesenchymal stem cell, *PGE1* prostaglandin E1

Flow cytometry revealed that MSC apoptosis increased in response to treatment with 200 μM H_2_O_2_. PGE1 reduced the apoptosis of MSCs when administered at 10 ng/mL, whereas 100 μM YC-1 reversed the protective effect of PGE1 (Fig. 3A; Additional file 1: Fig. S1). The WB experiment showed that the expression of Bcl-2 in MSCs decreased in response to treatment with H_2_O_2_. PGE1 increased HIF-1α expression, and YC-1 blocked this effect by inhibiting HIF-1α (Fig. 3B). To summarize, PGE1 increased the expression of Bcl-2 and reduced the apoptosis of MSCs by activating the HIF-1α pathway.

### CXCR4 expression on the surface of MSCs and MSC migration

**Fig. 4:**
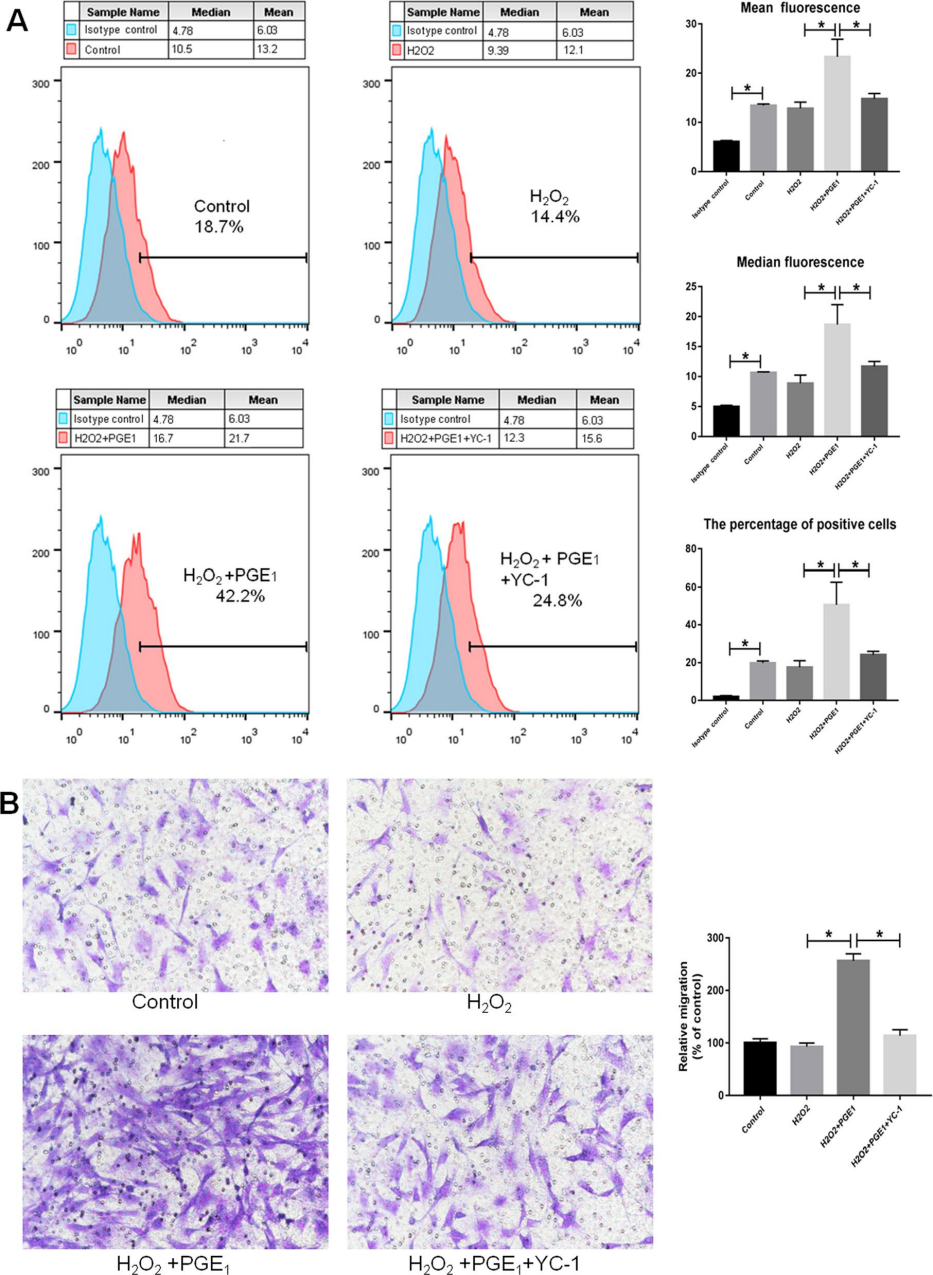
**A** As observed using flow cytometry, PGE1 increased the protein levels of CXCR4 on the surface of MSCs, an effect that was blocked by YC-1. **B** PGE1 increased SDF-1α-induced MSC migration in the Transwell migration assay, an effect that was blocked by YC-1 (*P* < 0.05). *MSC* mesenchymal stem cell, *PGE1* prostaglandin E1, *SDF-1α* stromal cell-derived factor 1 alpha

The results of the flow cytometry and Transwell migration assays showed that H_2_O_2_ treatment inhibited the expression of CXCR4 on the surface of MSCs and SDF-1α-induced MSC migration; however, there were no statistically significant differences. PGE1 increased the expression of CXCR4 on the surface of MSCs and MSC migration, with both effects being blocked by YC-1. This finding indicates that PGE1 increased the expression of CXCR4 on the surface of MSCs and promoted MSC migration through the HIF pathway (Fig. 4; Additional file 2: Fig. S2).

### PGE1 enhances VEGF secretion from MSCs through the HIF pathway

As observed using ELISA, H_2_O_2_ treatment marginally suppressed VEGF secretion from MSCs without any statistically significant difference. PGE1 increased VEGF secretion from MSCs, whereas YC-1 reversed this effect.

**Fig. 5:**
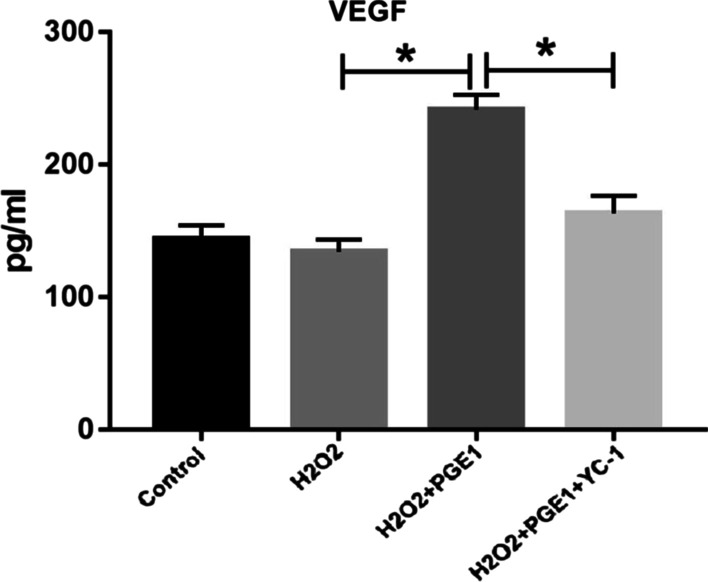
PGE1 increased VEGF secretion from MSCs, which was blocked by YC-1 (*P* < 0.05). *MSC* mesenchymal stem cell, *PGE1* prostaglandin E1, *VEGF* vascular endothelial growth factor

This indicates that PGE1 increased VEGF secretion from MSCs through the HIF pathway (Fig. 5; Additional file 3: Fig. S3).

### Serum MDA levels in rats

**Fig. 6:**
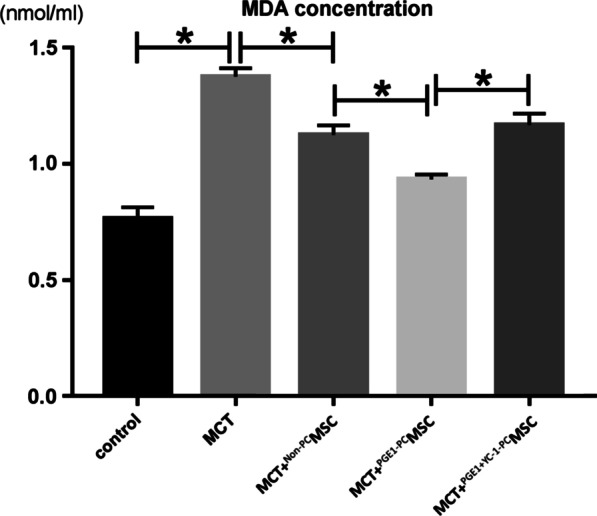
Serum MDA levels increased in response to MCT injection and reduced upon the transplantation of PGE1-preconditioned MSCs (*P* < 0.05). *MCT* monocrotaline, *MDA* methane dicarboxylic aldehyde, *MSC* mesenchymal stem cell, *PGE1* prostaglandin E1

ELISA using rat serum samples showed that the MDA levels increased 1 week after the MCT injection, which reflected the considerably high levels of oxidative stress in rats with PAH. The MDA levels decreased slightly in rats transplanted with non-preconditioned MSCs, which indicated a reduction in MCT-induced oxidative stress in vivo. Moreover, the MDA levels decreased significantly in rats transplanted with PGE1-preconditioned MSCs, meaning that the latter reduced the MCT-induced oxidative stress in vivo more potently than non-preconditioned MSCs. In rats transplanted with PGE1 + YC-1-preconditioned MSCs, the protective effects of PGE1 were reversed (Fig. 6).

## MSC homing to rat lungs

**Fig. 7:**
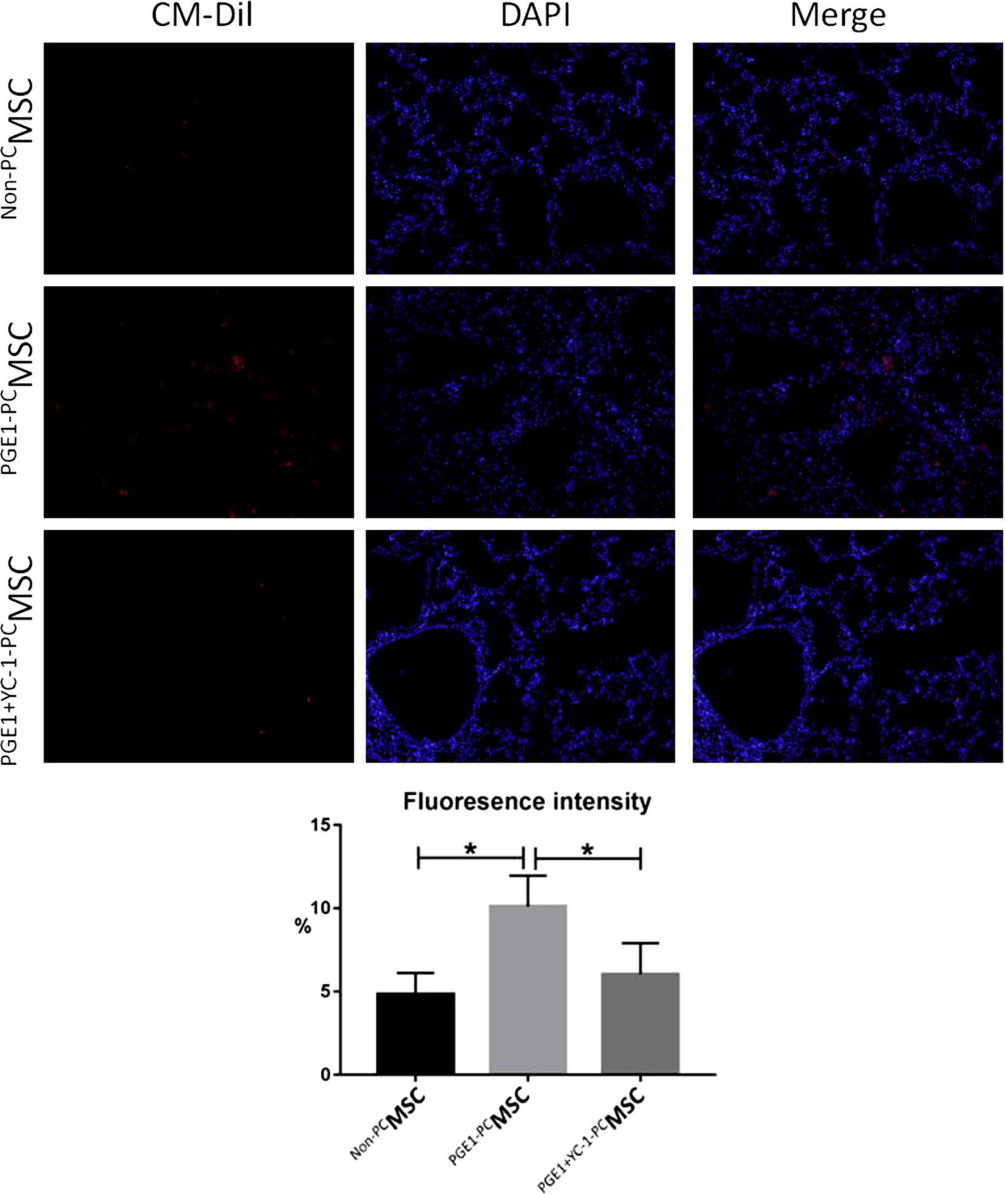
The proportion of MSCs homing to the lungs in the MCT + ^PGE1-PC^MSC group was higher than that in the MCT + ^PGE1+YC-1-PC^ MSC and MCT + ^Non-PC^MSC groups (*P* < 0.05). *MCT* monocrotaline, *MSC* mesenchymal stem cell, *Non-PC* non-preconditioned, *PGE1-PC* prostaglandin E1-preconditioned, *PGE1* + *YC-1* prostaglandin E1-preconditioned plus YC-1-treated

The proportion of MSCs that exhibited homing to lung tissues was estimated using fluorescence microscopy. Cells emitting red fluorescence were homing MSCs, whereas those emitting blue fluorescence were lung cells. The proportion of homing MSCs was the highest in the MCT + ^PGE1-PC^MSC group, while that in the MCT + ^PGE1+YC-1-PC^ MSC group was similar to that in the MCT + ^Non-PC^MSC group (Fig. 7).

### Mean pulmonary artery pressure, pulmonary artery systolic pressure, and right ventricular hypertrophy index in rats

**Fig. 8:**
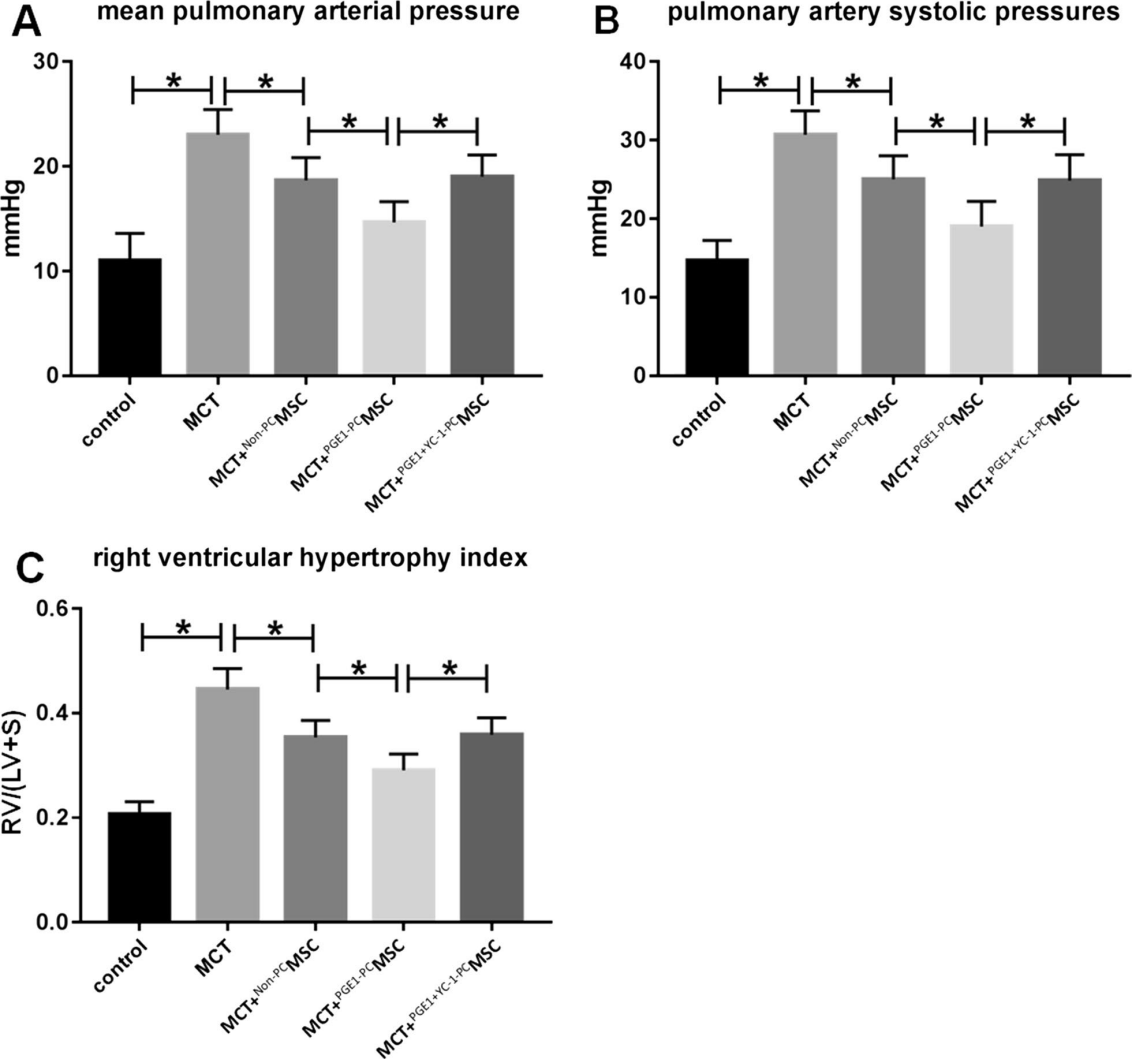
**A** Mean pulmonary artery pressure of rats. **B** Pulmonary artery systolic pressure of rats. **C** Right ventricular hypertrophy index of rats (*P* < 0.05)

The mean pulmonary artery pressure, pulmonary artery systolic pressure, and right ventricular hypertrophy index increased significantly in the control group. These parameters decreased marginally in the MCT + ^Non-PC^MSC group and significantly in the MCT + ^PGE1-PC^MSC group. The parameters obtained in the MCT + ^PGE1+YC-1-PC^MSC group were similar to those obtained in the MCT + ^Non-PC^MSC group (Fig. 8).

### %WT and %WA of rat pulmonary arterioles

**Fig. 9:**
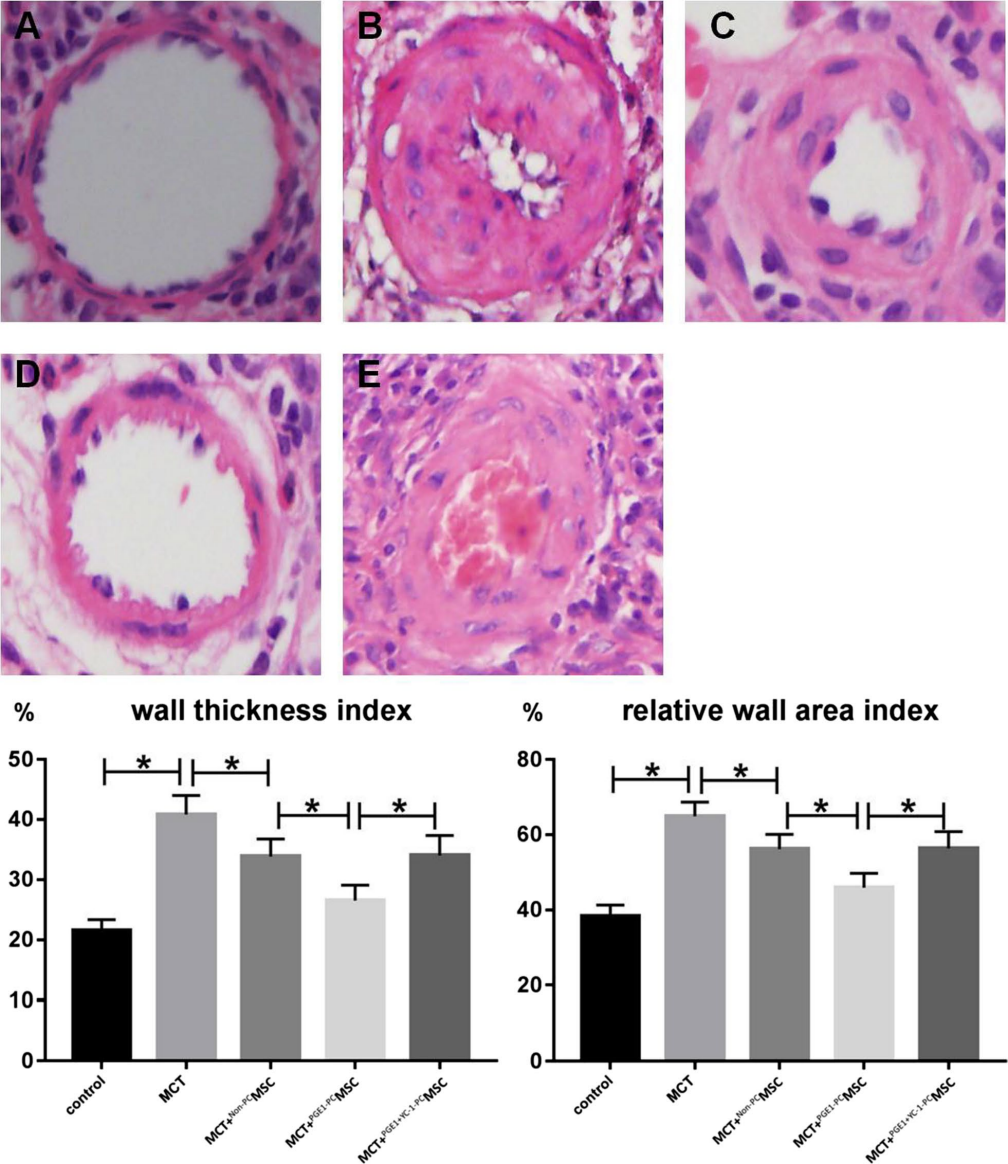
%WT and %WA of rats from the five groups. **A** Control group. **B** MCT group. **C** MCT + ^Non-PC^MSC group. **D** MCT + ^PGE1-PC^MSC group. **E** MCT + ^PGE1+YC-1-PC^MSC group (*P* < 0.05). *MCT* monocrotaline, *MSC* mesenchymal stem cell, *Non-PC* non-preconditioned, *PGE1-PC* prostaglandin E1-preconditioned, *PGE1* + *YC-1* prostaglandin E1-preconditioned plus YC-1-treated, *%WA* relative wall area index, *%WT* wall thickness index

Lung tissue sections were subjected to HE staining, and the %WT and %WA of pulmonary arterioles were calculated. These parameters increased significantly in the control group but were slightly lower in the MCT + ^Non-PC^MSC group and decreased significantly in the MCT + ^PGE1-PC^MSC group. The parameters obtained in the MCT + ^PGE1+YC-1-PC^MSC group were similar to those obtained in the MCT + ^Non-PC^MSC group (Fig. 9).

## Discussion

PAH is a difficult disease to treat, as traditional treatment methods mostly exhibit limited effectiveness. The application of stem cell therapy to PAH treatment is a novel approach [4]. Clinical trials have shown that the transplantation of endothelial progenitor cells (EPCs) can exert beneficial effects on the exercise ability and pulmonary hemodynamics of patients with PAH [21, 22]. At present, there are no ongoing clinical trials on MSC treatment. However, the therapeutic potential of MSCs has been demonstrated in preclinical studies. Compared with treatment with EPCs, treatment with MSCs is potentially advantageous and allows the allogeneic transplantation of cells without the need for immunosuppression. Therefore, MSCs are an attractive source of donor cells for PAH therapy [1].

The application of MSCs in PAH treatment is based on the homing of MSCs to the site of injury, differentiation into endothelial cells, repair of injured endothelial cells, and concurrent secretion of paracrine factors, including various cytokines and mediators (such as VEGF) that promote endothelial cell repair and angiogenesis, thereby improving the symptoms of PAH [1, 23]. Here, we tested a novel strategy for reducing apoptosis after MSC implantation, improving the homing rate of MSCs, and increasing the production of cytokines, including VEGF, which would help to treat PAH.

HIF-1α and its target genes are associated with apoptosis, cell migration, and secretion of various factors from cells. MSCs subjected to hypoxia and expressing increased levels of HIF-1α exhibited higher cell migration and transplant survival rates than untreated MSCs [15]. The pharmacological stabilization of HIF-1α can increase CXCR4 secretion and enhance the homing and implantation of stem cells [24]. *VEGF* is an important target gene of HIF-1α; therefore, HIF-1α activation increases VEGF expression [25, 26].

The use of PGE1, a prostaglandin commonly produced in the human body, was approved by the Food and Drug Administration in 1981 for the treatment of infants with catheter-dependent heart disease (CHD) who require the maintenance of an unobstructed catheter until palliative or corrective surgery can be performed. PGE1 is often used in newborns diagnosed with CHD immediately after birth [27].

It was shown that treatment with MSCs and PGE1 in rats significantly reduced apoptosis induced by serum deprivation, decreased the protein levels of Bax and caspase-3, and increased that of Bcl-2; however, the underlying mechanism remained unclear [28]. Prostaglandin E2 (PGE2) can increase HIF-1α expression in hematopoietic stem/progenitor cells (HSPCs), thereby increasing CXCR4 expression and promoting the homing of HSPCs [29]. Treatment of human prostate cancer cells with PGE2 enhanced VEGF expression by regulating HIF-1α expression [30].

We speculated that PGE1 may affect the function of MSCs through the HIF pathway. Using ELISA, we confirmed that the serum MDA concentration increased significantly 1 week after MCT injection, which indicated that the oxidative stress level in rats with PAH was considerably high. Oxidative stress is closely related to many heart diseases, such as atherosclerosis and clinical aortic valve disease [31].

Furthermore, we used 200 μM H_2_O_2_ to simulate oxidative stress in vivo [20], and MSC apoptosis increased observably upon this treatment. The expression of Bcl-2 in MSCs was low under oxidative stress. Transplantation of PGE1-pretreated MSCs activated the HIF-1α pathway, increased Bcl-2 expression, and suppressed MSC apoptosis. Our hypothesis was further validated when treatment with YC-1 suppressed the protective effects of PGE1-preconditioned MSCs.

After MSC transplantation, the number of MSCs homing to injured tissues is considerably small, and the percentage of MSCs detected in the lungs 21 days after transplantation is estimated to be 0.25%–0.375% of the MSCs initially administered; the homing of a low number of MSCs severely affects the functions of MSCs [12]. In this experiment, the ^Non-PC^MSC group exhibited low expression of intracellular and cell surface CXCR4, and only a few cells underwent SDF-1α-induced migration in vitro. In vivo experiments in the rats indicated that only a small number of non-preconditioned MSCs exhibited homing to the lung tissue. PGE1 treatment increased CXCR4 expression on the MSC surface and SDF-1α-induced MSC migration, whereas YC-1 treatment suppressed the aforementioned effects. This indicates the role of the HIF pathway in the protective effects of PGE1 on MSCs. Moreover, MSCs can secrete VEGF, which plays an important role in the treatment of PAH [13]. PGE1 preconditioning increased VEGF secretion in these cells through the HIF-1α pathway.

In vivo experiments in the rats indicated that the therapeutic effects of PGE1-preconditioned MSC transplantation were better than those of the non-preconditioned MSC transplantation. Moreover, the pulmonary artery systolic pressure, mean pulmonary artery pressure, right ventricular hypertrophy index, %WT, and %WA were lower in rats transplanted with PGE1-preconditioned MSCs than in the other groups.

## Conclusions

In our study, we confirmed that the pretreatment of MSCs with PGE1 can activate the HIF-1α pathway, reduce MSC apoptosis, and increase MSC migration to the site of injury and VEGF secretion. Rats implanted with PGE1-preconditioned MSCs experienced better therapeutic effects than those implanted with non-preconditioned MSCs. Our study indicated that PGE1 preconditioning can be used to improve the therapeutic potential of MSCs in PAH.

## Supplementary Information

**Additional file 1. Fig. S1**: Results of the flow cytometry experiment showing that MSC apoptosis increases in response to treatment with H2O2. PGE1 treatment reduces MSC apoptosis, whereas YC-1 blocks the protective effect of PGE1.

**Additional file 2. Fig. S2**: PGE1 increases SDF-1α-induced MSC migration in the Transwell migration assay; this effect was blocked by YC-1 treatment.

**Additional file 3. Fig. S3**: PGE1 increases VEGF secretion from MSCs, which was blocked by YC-1 treatment.

## Data Availability

All the data generated or analyzed during this study are included in this published article.

## Abbreviations

PAH: Pulmonary arterial hypertension
MSCs: Mesenchymal stem cells
VEGF: Vascular endothelial growth factor
HIF: Hypoxia-inducible factor
HIF-1α: HIF 1 alpha
YC-1: Lificiguat
Bcl-2: B-cell lymphoma 2
SDF-1α: Stromal cell-derived factor 1 alpha
CXCR4: C-X-C chemokine receptor 4
PGE1: Prostaglandin E1
FBS: Fetal bovine serum
PBS: Phosphate-buffered saline
CM-Dil: Chloromethylbenzamido dialkylcarbocyanine
MCT: Monocrotaline
MDA: Methane dicarboxylic aldehyde
ELISA: Enzyme-linked immunosorbent assay
HE: Hematoxylin and eosin
FITC: Fluorescein isothiocyanate
PE: Phycoerythrin
WB: Western blotting
BSA: Bovine serum albumin
EPCs: Endothelial progenitor cells
CHD: Catheter-dependent heart disease
PGE2: Prostaglandin E2
HSPCs: Hematopoietic stem/progenitor cells
α-MEM: Alpha-Minimum essential medium
