# Supplementary material for: Prostaglandin E1 reduces apoptosis and improves the homing of mesenchymal stem cells in pulmonary arterial hypertension by regulating hypoxia-inducible factor 1 alpha
Source: Stem Cell Res Ther. 2022 Jul 16;13:316. doi: 10.1186/s13287-022-03011-x (PMC9288720; doi:10.1186/s13287-022-03011-x)
Supplement: Supplementary file 1 — Additional file 1. Fig. S1: Results of the flow cytometry experiment showing that MSC apoptosis increases in response to treatment with H2O2. PGE1 treatment reduces MSC apoptosis, whereas YC-1 blocks the protective effect of PGE1. [file 13287_2022_3011_MOESM1_ESM.docx]

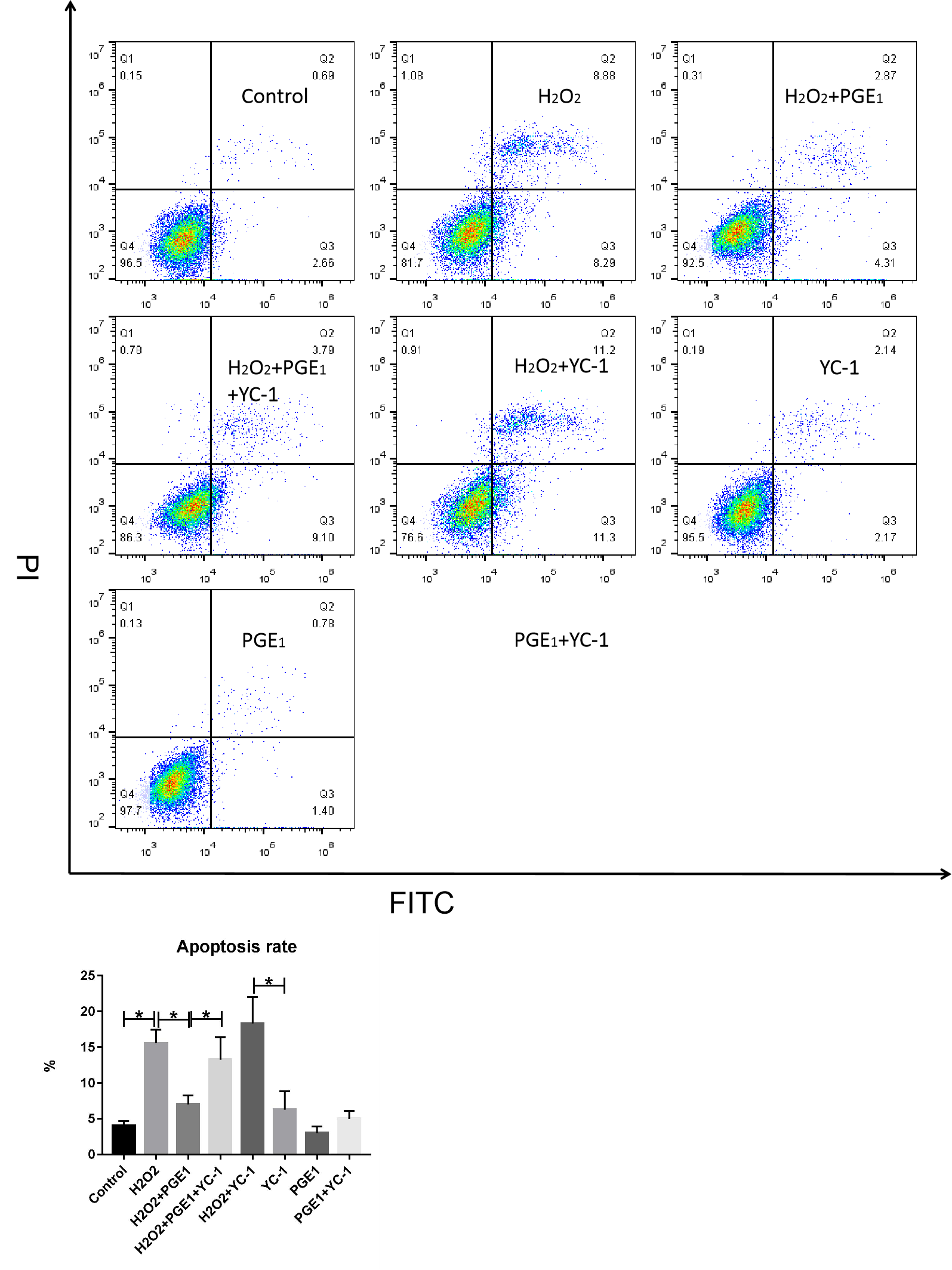


Supplemental Fig. 1 Results of the flow cytometry experiment showing that MSC apoptosis increases in response to treatment with H_2_O_2_. PGE1 treatment reduces MSC apoptosis, whereas YC-1 blocks the protective effect of PGE1.
