# Supplementary material for: Prostaglandin E1 reduces apoptosis and improves the homing of mesenchymal stem cells in pulmonary arterial hypertension by regulating hypoxia-inducible factor 1 alpha
Source: Stem Cell Res Ther. 2022 Jul 16;13:316. doi: 10.1186/s13287-022-03011-x (PMC9288720; doi:10.1186/s13287-022-03011-x)
Supplement: Supplementary file 2 — Additional file 2. Fig. S2: PGE1 increases SDF-1α-induced MSC migration in the Transwell migration assay; this effect was blocked by YC-1 treatment. [file 13287_2022_3011_MOESM2_ESM.docx]

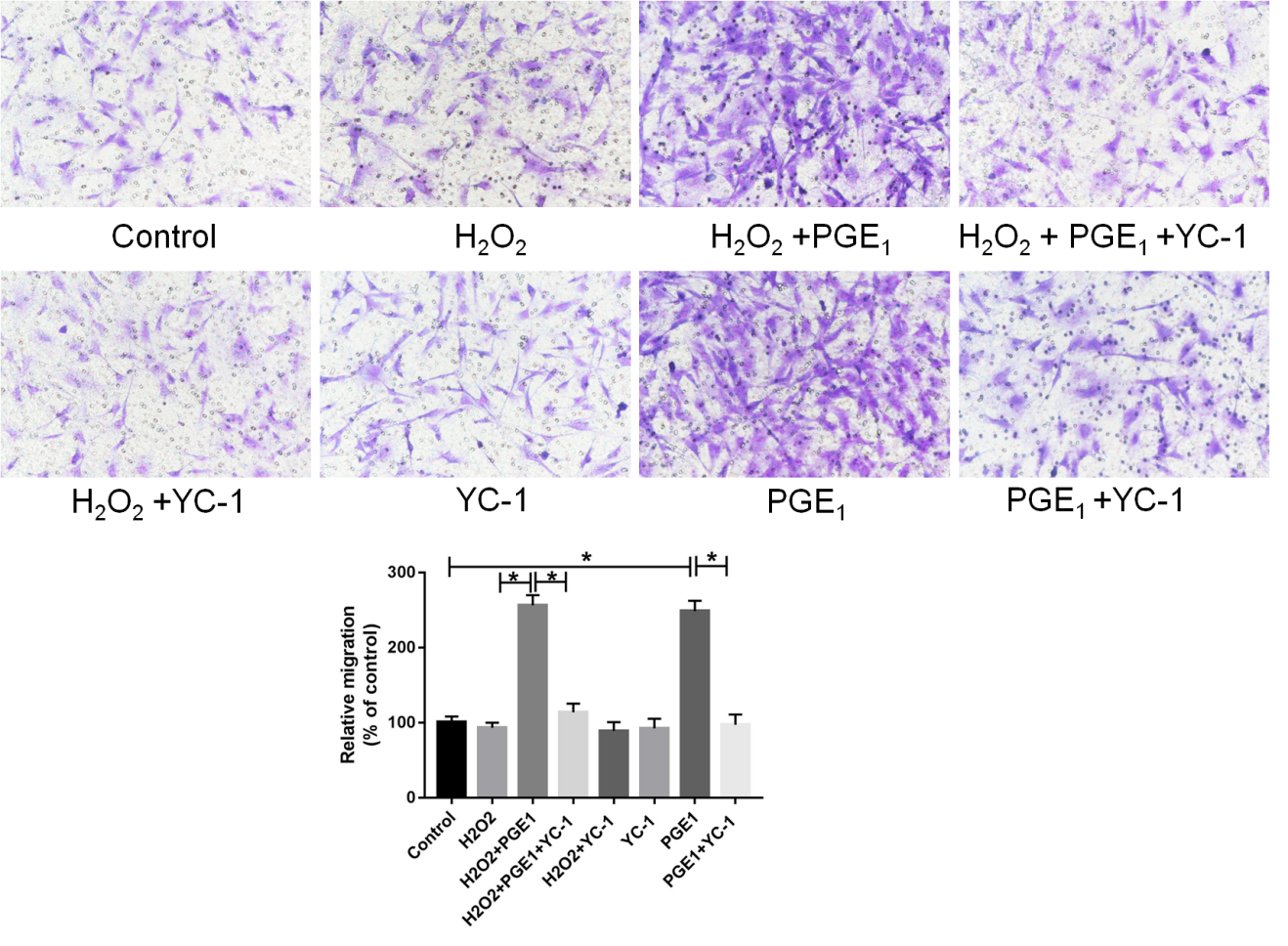


Supplemental Fig. 2 PGE1 increases SDF-1α-induced MSC migration in the Transwell migration assay; this effect was blocked by YC-1 treatment.
