# Supplementary figures and images for: Prostaglandin E1 reduces apoptosis and improves the homing of mesenchymal stem cells in pulmonary arterial hypertension by regulating hypoxia-inducible factor 1 alpha
Source: Stem Cell Res Ther. 2022 Jul 16;13:316. doi: 10.1186/s13287-022-03011-x (PMC9288720; doi:10.1186/s13287-022-03011-x)

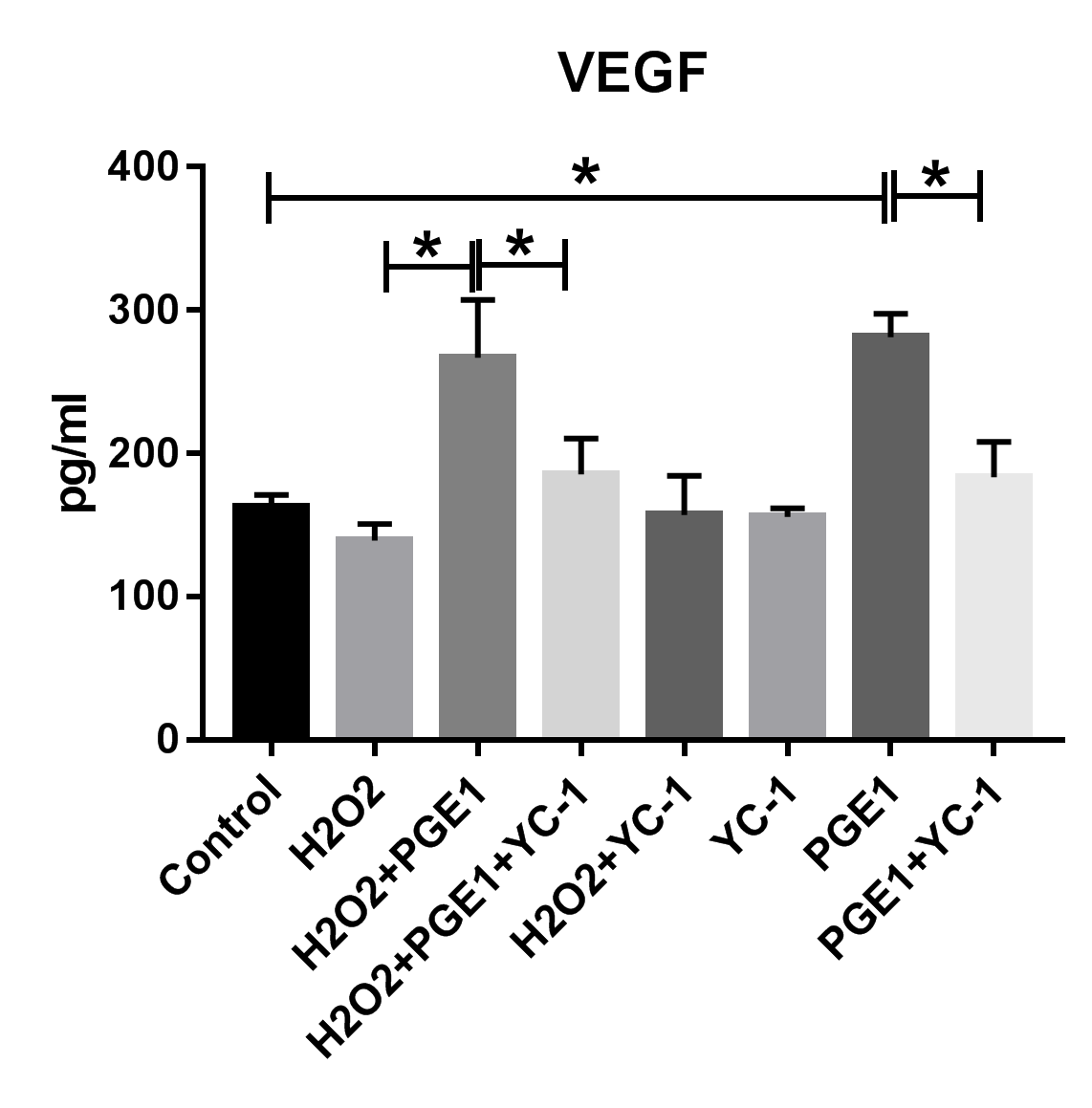


Supplemental Fig. 3 PGE1 increases VEGF secretion from MSCs, which was blocked by YC-1 treatment.

Supplement: Supplementary file 3 — Additional file 3. Fig. S3: PGE1 increases VEGF secretion from MSCs, which was blocked by YC-1 treatment. [file 13287_2022_3011_MOESM3_ESM.docx]
